# Radiological pattern in ARDS patients: partitioned respiratory mechanics, gas exchange and lung recruitability

**DOI:** 10.1186/s13613-021-00870-0

**Published:** 2021-05-17

**Authors:** Silvia Coppola, Tommaso Pozzi, Martina Gurgitano, Alessandro Liguori, Ejona Duka, Francesca Bichi, Arianna Ciabattoni, Davide Chiumello

**Affiliations:** 1 Department of Anesthesia and Intensive Care, ASST Santi Paolo E Carlo, San Paolo University Hospital, Milan, Italy; 2 Department of Health Sciences, University of Milan, Milan, Italy; 3 Division of Radiology, IEO European Institute of Oncology IRCCS, Milan, Italy; 4 Operative Unit of Radiology, Fondazione IRCCS Ca’ Granda Ospedale Maggiore Policlinico, Milan, Italy; 5 Radiologia Diagnostica Ed Interventistica, ASST Santi Paolo E Carlo, San Paolo University Hospital, Milan, Italy; 6 Coordinated Research Center On Respiratory Failure, University of Milan, Milan, Italy; 7 SC Anestesia E Rianimazione, ASST Santi Paolo E Carlo, Via Di Rudinì, Milan, Italy

**Keywords:** ARDS, Lung CT scan, Oxygenation, Driving pressure, Respiratory mechanics, Recruitment maneuver, PEEP

## Abstract

**Background:**

The ARDS is characterized by different degrees of impairment in oxygenation and distribution of the lung disease. Two radiological patterns have been described: a focal and a diffuse one. These two patterns could present significant differences both in gas exchange and in the response to a recruitment maneuver. At the present time, it is not known if the focal and the diffuse pattern could be characterized by a difference in the lung and chest wall mechanical characteristics. Our aims were to investigate, at two levels of PEEP, if focal vs. diffuse ARDS patterns could be characterized by different lung CT characteristics, partitioned respiratory mechanics and lung recruitability.

**Methods:**

CT patterns were analyzed by two radiologists and were classified as focal or diffuse. The changes from 5 to 15 cmH_2_O in blood gas analysis and partitioned respiratory mechanics were analyzed. Lung CT scan was performed at 5 and 45 cmH_2_O of PEEP to evaluate lung recruitability.

**Results:**

One-hundred and ten patients showed a diffuse pattern, while 58 showed a focal pattern. At 5 cmH_2_O of PEEP, the driving pressure and the elastance, both the respiratory system and of the lung, were significantly higher in the diffuse pattern compared to the focal (14 [11–16] vs 11 [9–15 cmH_2_O; 28 [23–34] vs 21 [17–27] cmH_2_O/L; 22 [17–28] vs 14 [12–19] cmH_2_O/L). By increasing PEEP, the driving pressure and the respiratory system elastance significantly decreased in diffuse pattern, while they increased or did not change in the focal pattern (*Δ*_15-5_: − 1 [− 2 to 1] vs 0 [− 1 to 2]; − 1 [− 4 to 2] vs 1 [− 2 to 5]). At 5 cmH_2_O of PEEP, the diffuse pattern had a lower lung gas (743 [537–984] vs 1222 [918–1974] mL) and higher lung weight (1618 [1388–2001] vs 1222 [1059–1394] g) compared to focal pattern. The lung recruitability was significantly higher in diffuse compared to focal pattern 21% [13–29] vs 11% [6–16]. Considering the median of lung recruitability of the whole population (16.1%), the recruiters were 65% and 22% in the diffuse and focal pattern, respectively.

**Conclusions:**

An early identification of lung morphology can be useful to choose the ventilatory setting. A diffuse pattern has a better response to the increase of PEEP and to the recruitment maneuver.

**Supplementary Information:**

The online version contains supplementary material available at 10.1186/s13613-021-00870-0.

## Background

According to the recent Berlin definition, the ARDS is defined as a non-cardiogenic pulmonary edema with different severity of hypoxemia [1]. However, due to the different etiology, time of onset, activation of inflammation, respiratory mechanics and lung recruitability, ARDS is a heterogeneous syndrome [2]. Consequently, different subgroups of patients (phenotypes) have been described with distinct clinical characteristics, response to the ventilatory treatment and outcome [3]. Another factor determining the heterogeneity could be the distribution of the disease into the lung [4]. Two different radiological patterns have been previously described: focal and not focal [5]. The focal pattern was defined at lung CT scan as a lobar distribution of attenuation in the lower part of the lung, while the diffuse/patchy pattern by a distribution of the attenuation throughout the lungs [5, 6]. Two recent studies reported in patients with non-focal ARDS pattern a higher plasma level of sRAGE, which is a marker of lung alveolar cell injury, with an associated higher hospital mortality [7, 8].

By applying a lung CT quantitative analysis, Rouby et al. showed in ARDS patients that the focal pattern had a higher end-expiratory lung gas volume and fraction of gas in the upper lobes compared to the not focal pattern [5]. By increasing PEEP up to 10 cmH_2_O the focal pattern showed a lower improvement in oxygenation, lung recruitability and higher overdistension both in the upper and lower lobes compared to not focal pattern [9, 10]. Furthermore, a CT scan performed during a recruitment maneuver at an airway pressure of 40 cmH_2_O in ARDS patients, demonstrated that the lung recruitability, defined as the ratio between the induced alveolar recruitment with zero end-expiratory positive pressure (PEEP), was 6% in the focal compared to 18% in the not focal pattern [6]. A recent randomized controlled trial evaluated if a mechanical ventilation strategy according to the focal and not focal pattern based on the use of low PEEP and high tidal volume compared to a high PEEP and low tidal volume could affect the outcome [11, 12]. The two groups received significantly different PEEP levels but the mortality was not different. However, up to 21% of the enrolled patients had received a wrong classification.

At the present time, it is not known whether the focal and the diffuse pattern could be characterized by a difference in the lung and chest wall mechanical characteristics, thus looking only at the airway pressure could be erroneous [13].

**Fig. 1:**
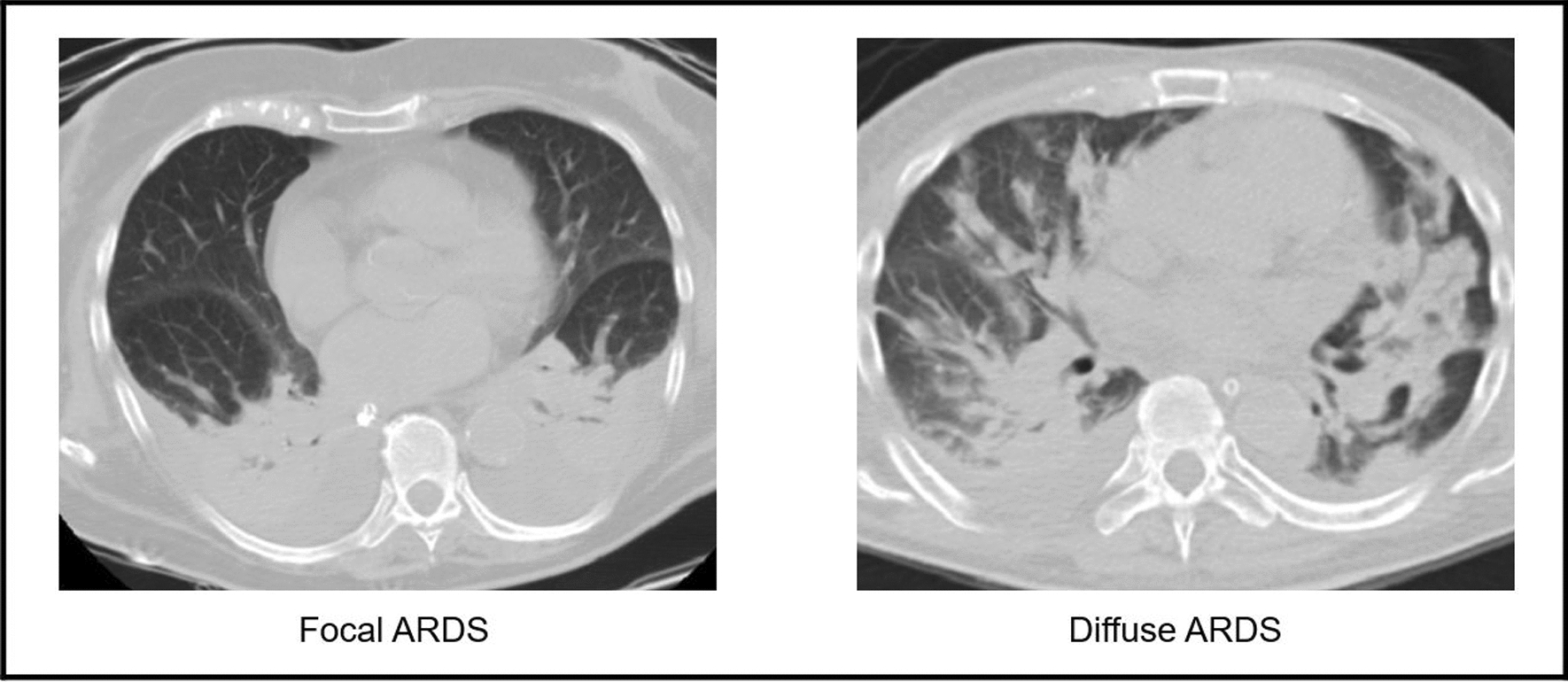
Two CT scans showing ARDS focal pattern on right panel and diffuse pattern on left panel

Our aims were to investigate whether different lung radiological ARDS morphology, focal vs. diffuse, could be characterized by different lung CT characteristics, partitioned respiratory mechanics, PEEP response and lung recruitability and to evaluate the possible differences in the focal and diffuse pattern according to the lung recruitability (Fig. 1).

## Methods

A total of 168 ARDS patients were retrospectively analyzed [14]. The institutional review board of each hospital approved the study and written consent was obtained according to the regulations applied in each institution.

At admission, patients were sedated, paralyzed and ventilated in volume control ventilation applying a tidal volume between 6 and 8 ml/Kg of ideal body weight with a PEEP value set by the attending physician to ensure an arterial saturation between 93 and 97%.

### Lung CT scan acquisition, morphological and quantitative analysis

Each patient was scanned twice by a Brightspeed 16-slice CT scanner (GE Medical Systems, Milwaukee, WI), from the lung apex to the diaphragm, during an end-expiratory pause at both 5 cmH_2_O and during an end-inspiratory pause at 45 cmH_2_O, using the following variables: 110 mAs, tube voltage 120 kV, rotation time 0.5 s, collimation 128 × 0.6 mm, pitch 0.85, reconstruction matrix 512 × 512 and 5 mm axial sections.

CT images were analyzed *a-posteriori* by two radiologists (M.G. and A.L.) blinded to patient history, on the Picture Archiving and Communication System workstation (Synapse PACS, FUJIFILM, Tokyo, Japan), using lung and mediastinal window level settings, with a width of 1500 and a level of – 500 HU.

Patterns of loss of aeration distribution were recognized as “focal” if consisting in areas of lung attenuation predominating in the lower lobes or gravitationally dependent parenchyma, “diffuse” if characterized by areas of lung attenuations widely distributed throughout the lungs, and “patchy” if there were lobar or segmental areas of lung attenuation in some parts of the lungs without anatomical limits. Patients showing a diffuse or patchy loss of aeration were classified as having a diffuse lung morphology [5, 6, 15].

Morphological assessment of lung attenuations was performed according to the Fleischner Society Nomenclature Committee [16], including CT consolidation and ground-glass opacification. Any disagreement between the two radiologists was resolved by the revision of a third blinded radiologist (E.D.).

For the quantitative analysis, the lung profiles of each CT scan slice were manually contoured, excluding hilar structures. Then, quantitative analysis was performed using a dedicated software (Maluna) [17] which computed the lung weight, lung gas volume, amount of over-inflated tissue (voxel density − 1000 to − 900 Hounsfield Units, HU), well-aerated tissue (− 899 to − 500 HU), poorly aerated tissue (− 499 to − 100 HU) and non-aerated tissue (− 100 to + 100 HU). Lung recruitability and overinflation were, respectively, computed as the ratio between the difference in non-aerated tissue at 5 cmH_2_O of PEEP and at 45 cmH_2_O of PEEP and the total lung tissue at 5 cmH_2_O of PEEP and as the ratio between the difference in well inflated tissue at 45 cmH_2_O of PEEP and at 5 cmH_2_O of PEEP and the total lung tissue at 5 cmH_2_O of PEEP [18].

### Respiratory mechanics

The esophageal pressure was measured using a standard balloon catheter (Smart Cath, Viasys, PalmSprings, USA) consisting of a tube 103 cm long with an external diameter of 3 mm and a thin-walled balloon 10 cm long. The esophageal catheter was emptied of air and introduced trans-orally into the esophagus to reach the stomach at a depth of 50–55 cm from the mouth. Subsequently the balloon was inflated with 1.5 ml of air. The intragastric position of the catheter was confirmed by a positive pressure deflection of intra-abdominal pressure during an external manual epigastric pressure. Subsequently, the catheter was retracted and positioned in the low esophageal position.

During an end-inspiratory and end-expiratory pause the airway and esophageal pressure were measured. The respiratory system, lung and chest wall elastance were computed according to the standard formulas:

- Respiratory system elastance (cmH_2_O/L) = (airway pressure at end-inspiratory pause − airway pressure at PEEP)/ tidal volume
- Chest wall elastance (cm H_2_O/L) = (esophageal pressure at end-inspiratory pause − esophageal pressure at PEEP)/tidal volume
- Lung elastance (cmH_2_O/L) = (Respiratory system elastance – Chest wall elastance)

### PEEP response

Before the PEEP trial, a recruitment maneuver was applied in pressure control ventilation with a PEEP of 5 cmH_2_O to reach 45 cmH_2_O with a respiratory rate of 10 for 2 min [18]. By maintaining constant the tidal volume, respiratory rate and oxygen fraction, 5 and 15 cmH_2_O of PEEP were applied. After 20 min at each PEEP level, respiratory mechanics measurements and blood gas analysis were performed.

The physiological dead space was computed according to the Enghoff modification of Bohr’s equation, with the mixed expired partial pressure of carbon dioxide being measured by a CO_2_SMO monitor (Novametrix, Wallingford, UK).

### Lung recruitability

Focal and diffuse pattern group were also divided in recruiters and non-recruiters, according to the median of lung recruitment of the whole population. Patients with a percentage of potentially recruitable of the total lung weight at or below or greater than the median value for the whole population were considered as non-recruiters or recruiters, respectively. Similarly, CT characteristics, gas exchange, respiratory mechanics and PEEP response were compared.

### Statistical analysis

Cohen’s *k* was calculated to assess the agreement between the radiologists in the diagnosis of the lung CT pattern.

Continuous data are presented as mean and standard deviation or median and interquartile range, as appropriate, while categorical data are reported as frequencies and percentages. Baseline characteristics of the patients with focal and diffuse pattern as well as the differences in respiratory mechanics, gas exchange and radiological data between two levels of airway pressures were compared by the Student *t* test or Mann–Whitney rank sum test, as appropriate. Two-way repeated measures analysis of variance (ANOVA) followed by all pairwise multiple comparison procedures (Holm–Sidak method) was applied to investigate the effect of the pattern and of the PEEP on respiratory mechanics, CT data, and gas exchanges; *p* values of 0.05 or less were considered statistically significant. The statistical analysis was done with SigmaPlot 11.0 (Systat Software, San Jose, CA) and RStudio (R Foundation for Statistical Computing, Vienna, Austria).

## Results

One-hundred and ten patients showed a diffuse pattern, while 58 patients showed a focal pattern. The interobserver agreement for the classification of the morphological pattern was evaluated as good (Cohen’s kappa equals to 0.75).

### ARDS characteristics

Table 1 shows the demographic and clinical characteristic of the ARDS patients according to the focal and diffuse pattern at lung CT scan at 5 cmH_2_O of PEEP. Lung CT scan was performed after 3 [1–5] and 2 [1–4] days from intubation in the focal and diffuse pattern, respectively (*p* = 0.175).

**Table 1:** Baseline characteristics at 5 cmH_2_O of PEEP in patients divided according to the radiological pattern

Characteristics	Focal group (*n* = 58)	Diffuse group (*n* = 110)	*p*
Age (years)	68 [60 to 76]	58 [44 to 69]	** < 0.001**
Male sex [% (n)]	72 (42)	70 (77)	0.882
BMI (kg/m^2^)	26 [23 to 29]	25 [22 to 28]	0.115
Origin of ARDS			**0.001**
Pulmonary [% (n)]	45 (26)	72 (79)	
Extrapulmonary [% (n)]	55 (32)	28 (31)	
ARDS severity			** < 0.001**
Mild [% (n)]	21 (12)	7 (8)	
Moderate [% (n)]	69 (40)	58 (64)	
Severe [% (n)]	10 (6)	35 (38)	
SAPS II	43.5 [33 to 58]	39 [32 to 51]	0.091
Tidal volume (mL)	523 [458 to 600]	484 [410 to 542]	**0.008**
Tidal volume per ideal body weight (mL/kg)	8.1 [7.0 to 9.2]	7.4 [6.7 to 8.3]	**0.003**
Respiratory rate (breath per minute)	15 [12 to 17]	16 [14 to 20]	**0.002**
Minute ventilation (L/min)	8.3 [7.5 to 8.8]	8.4 [7.0 to 9.9]	0.685
End-inspiratory airway pressure (cmH_2_O)	17 [14 to 20]	19 [16 to 21]	** < 0.001**
Driving pressure (cmH_2_O)	11 [9 to 15]	14 [11 to 16]	**0.001**
Respiratory system elastance (cmH_2_O/L)	21 [17 to 27]	28 [23 to 34]	** < 0.001**
Chest wall elastance (cmH_2_O/L)	6 [4 to 9]	5 [3 to 8]	0.185
Lung elastance (cmH_2_O/L)	14 [12 to 19]	22 [17 to 28]	** < 0.001**
PaCO_2_ (mmHg)	41 [37 to 45]	48 [42 to 53]	** < 0.001**
PaO_2_ (mmHg)	74 [65 to 86]	66 [58 to 75]	** < 0.001**
PaO_2_/FiO_2_	155 [126 to 187]	113 [84 to 147]	** < 0.001**
Physiological dead space	0.53 ± 0.10	0.66 ± 0.12	** < 0.001**

Continuous data are expressed as mean (SD) or median [interquartile range], while categorical data are expressed as % (number). Student *t* test or Mann–Whitney rank-sum test and Chi-square or Fisher exact test, were used as appropriate

Patients with focal pattern were significantly older but with similar SAPS II score. The diffuse group presented a higher percentage of pulmonary ARDS origin compared to focal group. The intensive care length of stay and mortality rate were not different between groups 19 [11–30] days vs 17 [10–30] days; 45% (49) vs 39% (22).

The diffuse group was ventilated with a significantly lower tidal volume but with similar minute ventilation compared to focal group. Arterial oxygenation was significantly lower, while physiological dead space and arterial carbon dioxide were significantly higher in the diffuse compared to focal pattern.

### Respiratory mechanics, lung stress and response to PEEP

At 5 cmH_2_O of PEEP the driving pressure and the elastance, both the respiratory system and of the lung, were significantly higher in the diffuse pattern (14 [11–16] vs 11 [9–15] cmH2O/L; 28 [23–34] vs 21 [17–27] cmH2O/L; 22 [17–28] vs 14 [12–19] cmH_2_O/L) (Table 1).

By increasing the PEEP from 5 to 15 cmH_2_O the amount of the changes in respiratory mechanics differed: the driving pressure and the elastance of respiratory system decreased significantly more in diffuse compared to focal pattern (Table 2).

**Table 2:** Changes in respiratory mechanics and gas exchange at 5 and 15 cmH_2_O of PEEP in patients divided according to the radiological pattern

Characteristics	Focal group (*n* = 58)	Diffuse group (*n* = 110)	*p*
Δ_15–5_ End-inspiratory airway pressure (cmH_2_O)	10 [8 to 11]	8 [7 to 11]	**0.022**
Δ_15–5_ Driving pressure (cmH_2_O)	0 [− 1 to 2]	− 1 [− 2 to 1]	**0.048**
Δ_15–5_ Respiratory system elastance (cmH_2_O/L)	1 [− 2 to 5]	− 1[− 4 to 2]	**0.008**
Δ_15–5_ Lung elastance (cmH_2_O/L)	1 [− 2 to 2]	− 1 [− 5 to 3]	0.061
Δ_15–5_ Chest Wall elastance (cmH_2_O/L)	1 ± 4	0 ± 3	0.231
Δ_15–5_ PaCO_2_ (mmHg)	0 [− 1 to 2]	0 [− 2 to 2]	0.321
Δ_15–5_ PaO_2_/FiO_2_	31 [3 to 49]	62 [31 to 106]	** < 0.001**
Δ_15–5_ Physiological dead space	0.02 ± 0.04	0.00 ± 0.04	**0.026**

Continuous data are expressed as mean (SD) or median [interquartile range]. Student *t* test or Mann–Whitney rank-sum test, were used as appropriate

The improvement in oxygenation and the reduction in the dead space was significantly higher (62 [31–106] vs 31 [3–49]) and lower (0.00 ± 0.04 vs 0.02 ± 0.04) in diffuse compared to focal pattern, respectively.

### CT scan characteristics and lung recruitment

At 5 cmH_2_O of PEEP, the diffuse pattern had a lower lung gas (743 [537–984] vs 1222 [918–1974] mL) and higher lung weight (1618 [1388–2001] vs 1222 [1059–1394] g) compared to focal pattern (Table 3). Similarly, the amount of not aerated tissue and well aerated tissue were higher (864 [561–1249] vs 464 [361–625] g) and lower (246 [185–334] vs 401 [288–492] g) in diffuse pattern compared to focal pattern, respectively (Table 3).

**Table 3:** Main computed tomography scan variables at 5 and 45 cmH_2_O of PEEP in patients divided according to the radiological pattern

Characteristics	Focal group (*n* = 58)	Diffuse group (*n* = 110)	*p*
Total lung gas (mL)	1222 [918 to 1974]	743 [537 to 984]	** < 0.001**
Total lung weight (g)	1222 [1059 to 1394]	1618 [1388 to 2001]	** < 0.001**
Total lung volume (mL)	2507 [2012 to 3224]	2481 [2124 to 3071]	0.985
Not aerated lung tissue (g)	464 [361 to 625]	864 [561 to 1249]	** < 0.001**
Poorly aerated lung tissue (g)	299 [217 to 364]	506 [376 to 691]	** < 0.001**
Well aerated lung tissue (g)	401 [288 to 492]	246 [185 to 334]	** < 0.001**
Over aerated lung tissue (g)	1 [0 to 6]	0 [0 to 1]	** < 0.001**
Δ_45–5_ total lung gas (mL)	1712 [1299 to 2262]	1268 [924 to 1799]	** < 0.001**
Δ_45–5_ total lung volume (mL)	1690 [1327 to 2338]	1287 [950 to 1805]	** < 0.001**
Δ_45–5_ not aerated lung tissue (g)	− 116 [− 193 to − 74]	− 355 [− 551 to − 190]	** < 0.001**
Δ_45–5_ poorly aerated lung tissue (g)	− 60 [− 128 to − 8]	11 [− 133 to 193]	**0.001**
Δ_45–5_ well aerated lung tissue (g)	133 [60 to 254]	343 [228 to 43]	** < 0.001**
Δ_45–5_ over aerated lung tissue (g)	51 [20 to 95]	6 [1 to 15]	** < 0.001**
Lung recruitment (%)	11 [6 to 16]	21 [13 to 29]	** < 0.001**
Overinflation (%)	4.1 [1.9 to 7.6]	0.3 [0.1 to 0.8]	** < 0.001**

Continuous data are expressed as mean (SD) or median [interquartile range]. Student *t* test or Mann–Whitney rank-sum test, were used as appropriate

Applying a recruitment maneuver, the lung recruitability and overinflation were significantly higher and lower in diffuse compared to focal pattern (21 [13–29] vs 11 [6–16] %; 0.3 [0.1–0.8] vs 4.1 [1.9–7.6] %).

### Recruiters and non-recruiters according to the morphological pattern

Considering the median of lung recruitability of the whole population (16.1%) to separate recruiters and non-recruiters, the recruiters were 65% and 22% in the diffuse and focal pattern, respectively. The recruiters in the diffuse pattern presented similar respiratory characteristics and lower oxygenation compared to not recruiters at 5 cmH_2_O of PEEP. (Additional file 1: Tables S4–S7). By increasing PEEP, the changes in respiratory mechanics and oxygenation were similar between the two groups (Additional file 1: Table S8). The total lung weight was similar, while the amount of not aerated tissue was significantly higher in recruiters compared to not recruiters. In the diffuse pattern, lung recruitability was (27 [22–34] vs 10 [8–14] %) in recruiters and not recruiters, respectively (Additional file 1: Tables S4, S10). Considering the focal pattern, the recruiters had a lower driving pressure and elastance, both the respiratory system and of the lung, with similar oxygenation compared to non-recruiters at 5 cmH_2_O of PEEP (Table 4, Additional file 1: Table S3). Similarly to the diffuse pattern, by increasing the PEEP the change in oxygenation was not different between groups (Additional file 1: Table S4). The total lung gas, the lung weight and the amount of not aerated tissue were similar between recruiters and not recruiters; however, after a recruitment maneuver from 5 to 45 cmH_2_O, the not aerated tissue significantly decreased more in recruiters than in non-recruiters (− 317 [− 432 to − 226] vs − 100 [− 131 to − 68] g). The lung recruitability was 24 [19–30] and 9 [6–11] % in recruiters and not recruiters, respectively (Additional file 1: Table S6).

**Table 4:** Gas exchange, respiratory mechanics and computed tomography scan variables within focal and diffuse group divided according to the radiological pattern

Characteristics	Recruiters	Non recruiters	*p*
*Focal ARDS group, n (%)*	13 (22)	45 (78)	
Driving Pressure (cmH_2_O)	9 [8 to 10]	13 [9 to 15]	**0.003**
PaO_2_/FiO_2_ (mmHg)	149 ± 40	165 ± 49	0.238
Δ_15–5_ Driving Pressure (cmH_2_O)	1 ± 1	0 ± 3	**0.048**
Δ_15–5_ PaO_2_/FiO_2_ (mmHg)	41 ± 48	32 ± 34	0.554
Total lung weight (g)	1158 [1074 to 1237]	1242 [1033 to 1398]	0.685
Overinflation (%)	6.5 [2.6 to 9.6]	3.8 [1.8 to 7.5]	0.304
Not aerated lung tissue (g)	503 [451 to 625]	434 [359 to 624]	0.208
*Diffuse ARDS group, n (%)*	71 (65)	39 (35)	
Driving Pressure (cmH_2_O)	14 ± 4	14 ± 4	0.659
PaO_2_/FiO_2_ (mmHg)	104 [75 to 139]	131 [106 to 168]	**0.001**
Δ_15–5_ Driving Pressure (cmH_2_O)	− 1 [− 2 to 1]	0 [− 2 to 1]	0.500
Δ_15–5_ PaO_2_/FiO_2_ (mmHg)	58 [32 to 108]	62 [30 to 95]	0.672
Total lung weight (g)	1660 [1381 to 2092]	1541 [1390 to 1948]	0.320
Overinflation (%)	0.3 [0.0 to 0.8]	0.5 [0.1 to 1.2]	0.223
Not aerated lung tissue (g)	977 [652 to 1287]	614 [437 to 967]	** < 0.001**

Continuous data are expressed as mean (SD) or median [interquartile range]. Student *t* test or Mann–Whitney rank-sum test, were used as appropriate

## Discussion

The major findings of this study enrolling 168 ARDS patients were: (1) at 5 cmH_2_O of PEEP the diffuse pattern had higher lung elastance with higher lung weight compared to focal pattern; (2) by increasing PEEP the diffuse pattern presented a higher increase in oxygenation and decrease in driving pressure and respiratory system elastance; (3) the lung recruitability and overinflation were higher and lower, respectively, in the diffuse pattern compared to the focal pattern; and (4) the recruiters in the diffuse group had lower oxygenation with higher amount of not aerated tissue, while in the focal group, they had similar oxygenation with lower driving pressure and elastance, with similar not aerated tissue compared to non-recruiters.

Since the first description of ARDS by Ashbaugh et al. in 1967 in a small group of patients, several subsequent definitions have been proposed [19–21]. Nowadays, the “Berlin definition” states that the ARDS is a syndrome with an acute onset with hypoxemia at different degree with bilateral pulmonary infiltrates not generated by cardiac failure or volume overload [1]. However, this definition has showed a low sensitivity and specificity when compared to the histhologic findings. To decrease the heterogeneity of the ARDS, Calfee et al. [2] by applying the latent class analysis and considering several clinical variables and biomarkers, computed at admission, identified two phenotypes. The hyperinflammatory phenotypes had a worse outcome and a more favourable response to higher PEEP compared to the hypoinflammatory. However, in these studies the lung morphology was not considered. Typically, the lung CT shows a heterogeneous pattern with normal regions, ground glass opacification and consolidations [22]. The ground glass opacification reflects the active inflammatory process in the interstitium and in the alveoli, while the consolidation is associated to a pulmonary parenchyma lesion in presence of exudate or transudate [23, 24]. In addition to the type of lung lesions, it has been suggested to evaluate the distribution of these in the lung accordingly to a focal or to non-focal pattern [25].

In fact, recent data showed that the lung morphology can be associated to the different phenotypes [7]. The diffuse pattern showed a different pathophysiology, with a more impairment of the alveolar fluid clearance, (an index of the resolution rate of alveolar oedema in ARDS), compared to the focal pattern [26]. The same group of authors also reported higher plasmatic levels of markers of lung alveolar type cell injury in the focal pattern compared to the diffuse pattern [7, 8].

In the present study, the morphological pattern was described by two independent radiologists by applying lung CT, considered the gold standard ARDS imaging technique, with a quite good agreement. In a recent study an incorrect classification of the morphological pattern was found in up to 21% of the patients, probably due to the use of chest X-ray which presented a lower accuracy and the absence of radiologist to classify the patterns.

Using the CT scan, in the present study, the focal and diffuse pattern were present in 34% and 65% of the all population, respectively, similarly in the previous studies, enrolling a lower number of ARDS patients, the diffuse pattern was present between in 65–70% of the patients [6, 25].

To better investigate the possible alterations in the lung and chest wall component of the respiratory system we estimated the changes in the pleural pressure by the measurement of the esophageal pressure [27]. At 5 cmH_2_O of PEEP, the higher respiratory system elastance in the diffuse pattern was due to the increase in the lung component, while the chest wall elastance was not different. This higher impairment of the lung mechanic was associated to a higher decrease in oxygenation in the diffuse pattern. In this group of patients also the lung gas volume was lower with a higher amount of not aerated lung tissue. Although not measured in the present study, the diffuse pattern has been found to be characterized by a higher lung inflammation which translates into a higher lung injury [7].

As known, the primary roles of PEEP are to improve the oxygenation and to stabilize the lung recruitment. The changes in oxygenation by increasing PEEP are mainly due to a decrease of the not aerated tissue (i.e., lung atelectasis) and to an improvement in the ventilation perfusion ratio [28]. In our study, the increasing of PEEP from 5 and 15 cmH_2_O, was associated to a significant difference in oxygenation among the two groups. The diffuse pattern had a significantly higher improvement in oxygenation compared to the focal. Similarly, in the study conducted by Rouby et al., the increase of PEEP from 0 to 10 cmH_2_O significantly improved the oxygenation in the diffuse compared to the lobar pattern [9, 10].

In addition, the changes in the respiratory system elastance and in the driving pressure were significantly higher in the diffuse pattern, both decreasing from 5 to 15 cmH_2_O of PEEP. Grasso et al. [29], considering only ARDS patients with lobar pattern, reported a higher increase in lung elastance, at higher levels of PEEP (13.2 ± 2.4 cmH_2_O) titrated according to the ARDSnet protocol compared to lower PEEP levels using a more personalized approach based on stress index strategy (6.8 ± 2.3 cmH_2_O).

Beside PEEP, the recruitment maneuvers could be part of the lung protective ventilation strategy which should ameliorate oxygenation and improve alveolar recruitment [30]. However, at the present time the role of recruitment maneuvers on long term outcome are still debatable [31]. To evaluate the effects of a recruitment maneuver in term of recruitment and overinflation, it was already showed that the CT remains the gold standard, while the use of respiratory variables computed at bedside has a low accuracy [18, 22]. Our group found that the lung recruitment in ARDS, computed as the decrease of not aerated tissue, was quite heterogeneous among ARDS patients and amounted to an average value of 13% [18].

In the current study, using the same definition, the average value of lung recruitment was 16,1%, with significantly different values between the focal and diffuse pattern (11 [6–16] vs 21 [13–29] %). Previous studies in ARDS patients showed that several variables such as the duration of ARDS, the type of recruitment maneuver applied, the severity at baseline and amount of fluid balance could explain the difference in lung recruitment [32]. The higher recruitment in the diffuse pattern could be explained by the higher amount of lung edema which represents tissue that can be re-opened [33] compared to the focal pattern [13]. Constantin et al. applying a similar recruitment maneuver to reach an airway pressure of 40 cmH_2_O, computing the alveolar recruitment as the decrease in the not aerated and poorly aerated lung volumes, showed a higher alveolar recruitment in the diffuse compared to the focal pattern (6% vs 18%) [6].

Concerning the overinflation during a recruitment maneuver, the diffuse pattern presented a lower amount although from a clinical point of view was quite negligible.

Comparing recruiters and not recruiters patients both within the diffuse and focal pattern, at 5 cmH_2_O of PEEP, the recruiters in the focal group had similar oxygenation, lower driving pressure and respiratory and lung elastance with similar radiological properties compared to non-recruiters except for the significant decrease of not aerated tissue after a recruitment maneuver, while in the diffuse group, the recruiters had lower oxygenation with similar respiratory characteristics and a higher amount of not aerated tissue. The response of PEEP was similar within the two patterns. These data suggest that in the diffuse pattern the excess tissue / edema is mainly localized in the interstitial space, while in the focal pattern is localized inside the pulmonary alveoli with different response to the recruitment maneuver.

Within each pattern, while the response to PEEP was similar in terms of oxygenation and mechanical properties between recruiters and non-recruiters, the decrease of not aerated lung tissue during a recruitment maneuver was significantly higher in recruiter patients.

Our study has a number of limitations. First, the feasibility of routine CT scan to evaluate lung recruitment. Recruitment maneuver cannot be considered part of lung protective ventilation independently of the CT characteristics of lung parenchyma because of the heterogeneity of this syndrome; however, the evaluation of lung recruitability using CT scan can be useful to titrate ventilation and reduce lung damage. Moreover, although the lung CT scan is not routinely used in ARDS due to the difficulty of patient transportation and risk radiation, it remains the gold standard lung imaging technique.

Second, the present study is a retrospective analysis of ARDS patients enrolled in the previous studies [14]; however, all the data analyzed and presented have never been explored and presented. Third, because the percentage of potentially recruitable lung is unknown and extremely variable in ARDS patients, we stratified recruiters and non-recruiters according the median value of the lung recruitability of our whole population, making this data not applicable for any study population.

## Conclusions

In conclusion an early identification of lung morphology can help to choose the mechanical ventilatory setting. A diffuse pattern is characterized by a higher lung weight and amount of not aerated tissue which better respond to higher PEEP levels and to the recruitment maneuver compared to focal pattern. However, within each radiological pattern just only the evaluation of the variation of not aerated tissue during a recruitment maneuver can be useful to identify recruiter from non-recruiter patients.

## Supplementary Information

**Additional file 1: Figure S1.** Study design and patient morphological characterization flow chart.** Table S1. **Characteristics of the study population at 5 cmH_2_O of PEEP. Continuous data are expressed as mean (SD) or median [interquartile range], while categorical data are expressed as % (number). Student *t* test or Mann–Whitney rank-sum test and Chi-square or Fisher exact test, were used as appropriate. **Table S2. **Respiratory mechanics, gas exchange and quantitative radiological characteristics at two levels of airways pressure in patients divided according to the radiological pattern. Two-way repeated measures ANalysis Of VAriance followed by all pairwise multiple comparison procedures (Holm–Šidák method) were used. **Table S3. **Focal population characteristics at PEEP 5 cmH_2_O according to the potential recruitment. Continuous data are expressed as mean (SD) or median [interquartile range], while categorical data are expressed as % (number). Student *t* test or Mann–Whitney rank-sum test and Chi-square or Fisher exact test, were used as appropriate. **Table S4.** Changes in respiratory mechanics and gas exchange at 2 levels of PEEP in focal group according to the potential recruitment. Continuous data are expressed as mean (SD) or median [interquartile range]. Student *t* test or Mann–Whitney rank-sum test, were used as appropriate. **Table S5. **Respiratory mechanics, gas exchange and quantitative radiological characteristics at two levels of airways pressure in FOCAL pattern group divided according to the potential recruitment. Two-way repeated measures ANalysis Of VAriance followed by all pairwise multiple comparison procedures (Holm–Šidák method) were used. **Table S6.** Quantitative radiological characteristics at PEEP 5 cmH_2_O and between two levels of airways pressure in the focal group according to the potential recruitment. Continuous data are expressed as mean (SD) or median [interquartile range]. Student *t* test or Mann–Whitney rank-sum test, were used as appropriate. **Table S7.** Diffuse population characteristics at PEEP 5 cmH_2_O according to the potential recruitment. Continuous data are expressed as mean (SD) or median [interquartile range], while categorical data are expressed as % (number). Student *t* test or Mann–Whitney rank-sum test and Chi-square or Fisher exact test, were used as appropriate. **Table S8.** Changes in respiratory mechanics and gas exchange at 2 levels of PEEP in diffuse group according to the potential recruitment. **Table S9. **Respiratory mechanics, gas exchange and quantitative radiological characteristics at two levels of airways pressure in DIFFUSE pattern group divided according to the potential recruitment. Two-way repeated measures ANalysis Of VAriance followed by all pairwise multiple comparison procedures (Holm–Šidák method) have been used for analysis. **Table S10. **Quantitative radiological characteristics at PEEP 5 cmH_2_O and between two levels of airways pressure in the diffuse group according to the potential recruitment. Continuous data are expressed as mean (SD) or median [interquartile range], while categorical data are expressed as % (number). Student *t* test or Mann–Whitney rank-sum test and Chi-square or Fisher exact test, were used as appropriate.

## Data Availability

The dataset used and analyzed during the current study are available from the corresponding author on reasonable request.

## Abbreviations

ARDS: Acute respiratory distress syndrome
CT: Computed tomography
PEEP: Positive end-expiratory pressure
