# Supplementary material for: Radiological pattern in ARDS patients: partitioned respiratory mechanics, gas exchange and lung recruitability
Source: Ann Intensive Care. 2021 May 17;11:78. doi: 10.1186/s13613-021-00870-0 (PMC8128955; doi:10.1186/s13613-021-00870-0)
Supplement: Supplementary file 1 — Additional file 1: Figure S1. Study design and patient morphological characterization flow chart. Table S1. Characteristics of the study population at 5 cmH2O of PEEP. Continuous data are expressed as mean (SD) or median [interquartile range], while categorical data are expressed as % (number). Student t test or Mann–Whitney rank-sum test and Chi-square or Fisher exact test, were used as appropriate. Table S2. Respiratory mechanics, gas exchange and quantitative radiological characteristics at two levels of airways pressure in patients divided according to the radiological pattern. Two-way repeated measures ANalysis Of VAriance followed by all pairwise multiple comparison procedures (Holm–Šidák method) were used. Table S3. Focal population characteristics at PEEP 5 cmH2O according to the potential recruitment. Continuous data are expressed as mean (SD) or median [interquartile range], while categorical data are expressed as % (number). Student t test or Mann–Whitney rank-sum test and Chi-square or Fisher exact test, were used as appropriate. Table S4. Changes in respiratory mechanics and gas exchange at 2 levels of PEEP in focal group according to the potential recruitment. Continuous data are expressed as mean (SD) or median [interquartile range]. Student t test or Mann–Whitney rank-sum test, were used as appropriate. Table S5. Respiratory mechanics, gas exchange and quantitative radiological characteristics at two levels of airways pressure in FOCAL pattern group divided according to the potential recruitment. Two-way repeated measures ANalysis Of VAriance followed by all pairwise multiple comparison procedures (Holm–Šidák method) were used. Table S6. Quantitative radiological characteristics at PEEP 5 cmH2O and between two levels of airways pressure in the focal group according to the potential recruitment. Continuous data are expressed as mean (SD) or median [interquartile range]. Student t test or Mann–Whitney rank-sum test, were used as appropriate. Table S7. [file 13613_2021_870_MOESM1_ESM.docx]

RADIOLOGICAL PATTERN IN ARDS PATIENTS: PARTITIONED RESPIRATORY MECHANICS, GAS EXCHANGE AND LUNG RECRUITABILITY

**Additional file**

**
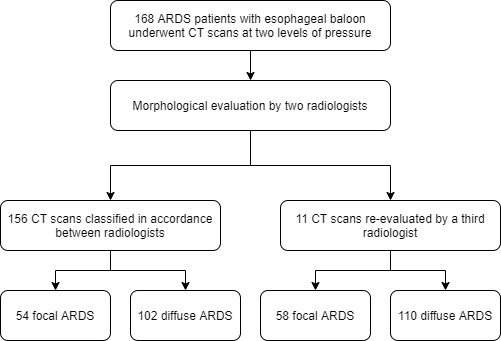
**

**Figure 1S. Study design and patient morphological characterization flow chart.**

| Characteristics | STUDY POPULATION  (n=168) |
| --- | --- |
| Age (years) | 62 [48 – 72] |
| Male sex [% (number)] | 71 (119) |
| BMI (kg/m^2^) | 25 [22 – 28] |
| Origin of ARDS  Pulmonary [% (n)]  Extrapulmonary [% (n)] | 62 (105)  38 (63) |
| ARDS severity  Mild [% (n)]  Moderate [% (n)]  Severe [% (n)] | 20 (12)  62 (104)  26 (44) |
| SAPS II | 40 [32 - 53] |
| Tidal volume (mL) | 504 ± 107 |
| Tidal volume per ideal body weight (mL/kg) | 7.6 [6.7 – 8.7] |
| Respiratory rate (breath for minute) | 15 [14 – 18] |
| Minute ventilation (L/min) | 8.3 [7.0 – 9.6] |
| End-inspiratory airway pressure (cmH_2_O) | 18 [16 – 21] |
| Driving pressure (cmH_2_O) | 13 [10 – 16] |
| Respiratory system elastance (cmH_2_O/L) | 26 [20 – 32] |
| Chest wall elastance (cmH_2_O/L) | 6 [4 – 9] |
| Lung elastance (cmH_2_O/L) | 19 [14 – 25] |
| PaCO_2_ (mmHg) | 45 [40 – 52] |
| PaO_2_ (mmHg) | 69 [61 – 79] |
| PaO_2_/FiO_2_ | 132 [100 – 167] |
| Physiological dead space | 0.61 ± 0.13 |
| ICU length of stay, *days* | 18 [10 – 30] |
| ICU mortality  Dead  Alive | 43 (71)  57 (94) |

**Table 1S. Characteristics of the study population at 5 cmH_2_O of PEEP. Continuous data are expressed as mean (SD) or median [interquartile range], while categorical data are expressed as % (number). Student t test or Mann-Whitney rank-sum test and chi-square or Fisher exact test, were used as appropriate.**

| Characteristics | FOCAL  (n=58) | DIFFUSE  (n=110) | *p_GROUP_* | *p_PEEP_* | *p_GROUP_*  *_x PEEP_* |
| --- | --- | --- | --- | --- | --- |
| End-inspiratory airway pressure (cmH_2_O)  PEEP 5 cmH_2_O  PEEP 15 cmH_2_O | 17 [14 – 20]  26 [24 – 29]^§^ | 19 [16 – 21]  28 [25 – 30]^§^ | *0.181* | ***<0.001*** | ***0.046*** |
| Driving Pressure (cmH_2_O)  PEEP 5 cmH_2_O  PEEP 15 cmH_2_O | 12 [9-15]  12 [10-15] | 14 [11-16]  13 [11-16] | *0.124* | *0.911* | *0.085* |
| Respiratory system elastance (cmH_2_O/L)  PEEP 5 cmH_2_O  PEEP 15 cmH_2_O | 21 [18-27]  23 [18-28] | 28 [23-34]^°^  27 [21-33] | *0.146* | *0.588* | ***0.032*** |
| Lung elastance (cmH_2_O/L)  PEEP 5 cmH_2_O  PEEP 15 cmH_2_O | 15 [13-19]  16 [14-19] | 22 [17-28]^°^  21 [17-26]^°^ | ***<0.001*** | *0.886* | *0.165* |
| Chest wall elastance (cmH_2_O/L)  PEEP 5 cmH_2_O  PEEP 15 cmH_2_O | 6 [5-8]  7 [5-10]^§^ | 6 [4-8]  6 [4-8]^°^ | ***0.046*** | ***0.014*** | *0.231* |
| Stress, (cmH_2_O)  PEEP 5 cmH_2_O  PEEP 15 cmH_2_O | 11.5 [9.2 – 15.2]  18.3 [16.2 – 20.3] | 14.3 [12.0 – 16.9]  20.8 [18.2 – 23.5] | ***<0.001*** | ***<0.001*** | *0.665* |
| Absolute ei transpulmonary pressure, (cmH_2_O)  PEEP 5 cmH_2_O  PEEP 15 cmH_2_O | -0.8 [-2.1 – 2.1]  5.5 [3.9 – 9.9] | 1.3 [-0.8 – 4.9]  7.9 [5.4 – 11.4] | ***0.004*** | ***<0.001*** | *0.617* |
| PaCO_2_ (mmHg)  PEEP 5 cmH_2_O  PEEP 15 cmH_2_O | 41 [37-45]  42 [37-46] | 48 [42-53]^°^  47 [42-54]^°^ | ***<0.001*** | *0.147* | *0.416* |
| PaO_2_/FiO_2_ (mmHg)  PEEP 5 cmH_2_O  PEEP 15 cmH_2_O | 155 [126-187]  195 [149-231]^§^ | 113 [84-146]^°^  178 [143-242]^§^ | ***0.030*** | ***<0.001*** | ***<0.001*** |
| Physiological dead space  PEEP 5 cmH_2_O  PEEP 15 cmH_2_O | 0.55 [0.47-0.61]  0.57 [0.51-0.61]^§^ | 0.67 [0.60-0.73]^°^  0.64 [0.59-0.72]^°^ | ***<0.001*** | ***0.005*** | ***0.026*** |
| Total lung gas (mL)  PEEP 5 cmH_2_O  PEEP 45 cmH_2_O | 1222 [923-1951]  3050 [2381-3933]^§^ | 743 [538-984]^°^  2151 [1555-2680]^§°^ | ***<0.001*** | ***<0.001*** | ***<0.001*** |
| Total lung weight (g)  PEEP 5 cmH_2_O  PEEP 45 cmH_2_O | 1222 [1061-1388]  1221 [1026-1404] | 1618 [1389-2001]^°^  1645 [1413-2007]^°^ | ***<0.001*** | *0.540* | *0.189* |
| Total lung volume (mL)  PEEP 5 cmH_2_O  PEEP 45 cmH_2_O | 2507 [2013-3212]  4198 [3653-5294]^§^ | 2481 [2133-3051]  3846 [3225-4605]^§°^ | *0.134* | ***<0.001*** | ***<0.001*** |
| Not aerated lung tissue (g)  PEEP 5 cmH_2_O  PEEP 45 cmH_2_O | 464 [362-624]  299 [228-461]^§^ | 864 [564-1249]^°^  473 [252-702]^§°^ | ***<0.001*** | ***<0.001*** | ***<0.001*** |
| Poorly aerated lung tissue (g)  PEEP 5 cmH_2_O  PEEP 45 cmH_2_O | 299 [218-362]  229 [181-273]^§^ | 506 [381-687]^°^  571 [339-758]^§°^ | ***<0.001*** | *0.417* | ***<0.001*** |
| Well aerated lung tissue (g)  PEEP 5 cmH_2_O  PEEP 45 cmH_2_O | 401 [289-488]  577 [455-672]^§^ | 246 [186-334]^°^  618 [478-735] ^§°^ | ***0.062*** | ***<0.001*** | ***<0.001*** |
| Over aerated lung tissue (g)  PEEP 5 cmH_2_O  PEEP 45 cmH_2_O | 1 [0-6]  53 [20-96]^§^ | 0 [0-0]  7 [1-17]^§°^ | ***<0.001*** | ***<0.001*** | ***<0.001*** |
| Not aerated lung tissue (%)  PEEP 5 cmH_2_O  PEEP 45 cmH_2_O | 41 [33-50]  28 [20-38]^§^ | 53 [40-63]^°^  28 [16-39]^§^ | ***0.011*** | ***<0.001*** | ***<0.001*** |
| Poorly aerated lung tissue (%)  PEEP 5 cmH_2_O  PEEP 45 cmH_2_O | 24 [20-27]  19 [16-22]^§^ | 32 [23-40]^°^  32 [25-41]^°^ | ***<0.001*** | ***0.005*** | ***<0.001*** |
| Well aerated lung tissue (%)  PEEP 5 cmH_2_O  PEEP 45 cmH_2_O | 34 [28-41]  47 [39-57]^§^ | 14 [10-22]^°^  35 [26-46]^§°^ | ***<0.001*** | ***<0.001*** | ***<0.001*** |
| Over aerated lung tissue (%)  PEEP 5 cmH_2_O  PEEP 45 cmH_2_O | 0 [0-1]  5 [2-8]^§^ | 0 [0-0]  0 [0-1]^§°^ | ***<0.001*** | ***<0.001*** | ***<0.001*** |

**Table 2S. Respiratory mechanics, gas exchange and quantitative radiological characteristics at two levels of airways pressure in patients divided according to the radiological pattern. Two-way repeated measures ANalysis Of VAriance followed by all pairwise multiple comparison procedures (Holm-Šidák method) were used.**

**§: significant post-hoc comparison with PEEP 5 cmH_2_O;**

**°: significant post-hoc comparison with FOCAL group.**

| FOCAL PATTERN | Recruiters  (n=13) | Non Recruiters  (n=45) | *p* |
| --- | --- | --- | --- |
| Age (years) | 72 [64 – 79] | 67 [60 – 75] | *0.115* |
| Male sex [% (number)] | 85 (11) | 69 (31) | *0.441* |
| Weight (kg) | 80 [70 – 82] | 74 [62 – 95] | *0.940* |
| BMI (kg/m^2^) | 23 [21 – 25] | 26 [23 – 30] | *0.661* |
| Origin of ARDS  Pulmonary [% (n)]  Extrapulmonary [% (n)] | 77 (10)  23 (3) | 36 (16)  64 (29) | ***0.020*** |
| ARDS severity  Mild [% (n)]  Moderate [% (n)]  Severe [% (n)] | 15 (2)  85 (11)  0 (0) | 22 (10)  64 (29)  14 (6) | *0.279* |
| SAPS II | 46 [34 – 54] | 43 [32 – 58] | *0.823* |
| Tidal volume (mL) | 485 ± 71 | 553 ± 115 | ***0.014*** |
| Tidal volume per ideal body weight (mL/kg) | 7.3 ± 1.0 | 8.6 ± 1.6 | ***0.001*** |
| Respiratory rate (breaths per minute) | 15 [14 – 16] | 15 [12 – 18] | *0.955* |
| Minute ventilation (L/min) | 8.3 [7.6 – 9.0] | 7.7 [7. – 9.9] | *0.323* |
| Plateau pressure (cmH_2_O) | 14 ± 3 | 18 ± 4 | ***<0.001*** |
| Driving pressure (cmH_2_O) | 9 [8 – 10] | 13 [9 – 15] | ***0.003*** |
| Respiratory system elastance (cmH_2_O/L) | 19 [16 – 20] | 22 [19 – 27] | ***0.044*** |
| Chest wall elastance (cmH_2_O/L) | 6 [4 - 9] | 6 [5 - 9] | *0.403* |
| Lung elastance (cmH_2_O/L) | 13 [10 - 15] | 16 [13 – 21] | ***0.027*** |
| PaCO_2_ (mmHg) | 43 [39 – 46] | 41 [36 – 45] | *0.412* |
| PaO_2_ (mmHg) | 68 [64 – 78] | 76 [68 – 86] | *0.288* |
| PaO_2_/FiO_2_ | 149 ± 40 | 165 ± 49 | *0.238* |
| Physiological dead space | 0.52 ± 0.06 | 0.53 ± 0.11 | *0.723* |
| ICU length of stay, days | 15 [8 – 17] | 20 [10 – 38] | ***0.037*** |
| ICU mortality  Dead  Alive | 46 (6)  54 (7) | 36 (16)  64 (28) | *0.755* |

**Table 3S. Focal population characteristics at PEEP 5 cmH_2_O according to the potential recruitment. Continuous data are expressed as mean (SD) or median [interquartile range], while categorical data are expressed as % (number). Student t test or Mann-Whitney rank-sum test and chi-square or Fisher exact test, were used as appropriate.**

| FOCAL PATTERN | Recruiters  (n=13) | Non Recruiters  (n=45) | *p* |
| --- | --- | --- | --- |
| Δ_15-5_ End-inspiratory airway pressure (cmH_2_O) | 11 ± 2 | 9 ± 3 | *0.061* |
| Δ_15-5_ Driving Pressure (cmH_2_O) | 1 ± 1 | 0 ± 3 | ***0.048*** |
| Δ_15-5_ Respiratory system elastance (cmH_2_O/L) | 3 ± 3 | 1 ± 6 | *0.090* |
| Δ_15-5_ Lung elastance (cmH_2_O/L) | 1 [1 – 3] | 0 [-3 – 2] | *0.068* |
| Δ_15-5_ Chest wall elastance (cmH_2_O/L) | 0 [-1 – 1] | 1 [-1 – 3] | *0.343* |
| Δ_15-5_ PaCO_2_ (mmHg) | 2 ± 3 | 0 ± 2 | *0.213* |
| Δ_15-5_ PaO_2_/FiO_2_ | 41 ± 48 | 32 ± 34 | *0.554* |
| Δ_15-5_ Physiological dead space | 0.03 ± 0.04 | 0.02 ± 0.04 | *0.524* |

**Table 4S. Changes in respiratory mechanics and gas exchange at 2 levels of PEEP in focal group according to the potential recruitment. Continuous data are expressed as mean (SD) or median [interquartile range]. Student t test or Mann-Whitney rank-sum test, were used as appropriate.**

| FOCAL PATTERN | Recruiters  (n=13) | Non Recruiters  (n=45) | *p_GROUP_* | *p_PEEP_* | *p_GROUP X PEEP_* |
| --- | --- | --- | --- | --- | --- |
| End-inspiratory airway pressure (cmH_2_O)  PEEP 5 cmH_2_O  PEEP 15 cmH_2_O | 14 [12 – 16]  25 [23 – 27] | 18 [15 – 20]  28 [25 – 30] | *1* | *0.124* | *0.916* |
| Driving Pressure (cmH_2_O)  PEEP 5 cmH_2_O  PEEP 15 cmH_2_O | 9 [8-10]  10 [9-11] | 13 [9-15]  12 [10-15] | *1* | *0.832* | *0.850* |
| Respiratory system elastance (cmH_2_O/L)  PEEP 5 cmH_2_O  PEEP 15 cmH_2_O | 19 [16-20]  21 [19-27] | 23 [19-27]  23 [18-28] | *1* | *0.581* | *0.753* |
| Lung elastance (cmH_2_O/L)  PEEP 5 cmH_2_O  PEEP 15 cmH_2_O | 13 [11-14]  16 [15-18] | 16 [13-20]  16 [14-19] | *0.082* | *0.114* | *0.088* |
| Chest wall elastance (cmH_2_O/L)  PEEP 5 cmH_2_O  PEEP 15 cmH_2_O | 5 [4-7]  6 [5-8] | 6 [5-8]  7 [6-10] | *0.326* | *0.134* | *0.579* |
| PaCO_2_ (mmHg)  PEEP 5 cmH_2_O  PEEP 15 cmH_2_O | 43 [39-46]  45 [41-47]^§^ | 41 [36-45]  41 [37-45] | *0.431* | ***0.019*** | *0.123* |
| PaO_2_/FiO_2_ (mmHg)  PEEP 5 cmH_2_O  PEEP 15 cmH_2_O | 146 [113-173]  195 [143 -220]^§^ | 162 [137-191]  194 [157-236]^§^ | *0.482* | ***<0.001*** | *0.471* |
| Physiological dead space  PEEP 5 cmH_2_O  PEEP 15 cmH_2_O | 0.53 [0.51-0.58]  0.57 [0.53-0.60] | 0.56 [0.47-0.63]  0.57 [0.48-0.61]^§^ | *0.907* | ***0.015*** | *0.575* |
| Total lung gas (mL)  PEEP 5 cmH_2_O  PEEP 45 cmH_2_O | 1266 [977-1538]  3372 [3039-4073]^§^ | 1192 [918-1983]  2877 [2297-3716]^§^ | *0.650* | ***<0.001*** | ***0.001*** |
| Total lung weight (g)  PEEP 5 cmH_2_O  PEEP 45 cmH_2_O | 1158 [1074-1237]  1115 [1033-1332] | 1242 [1033-1397]  1243 [994-1407] | *0.966* | *0.218* | *0.144* |
| Total lung volume (mL)  PEEP 5 cmH_2_O  PEEP 45 cmH_2_O | 2355 [2251-2775]  4691 [3948-5337]^§^ | 2574 [1997-3346]  4080 [3416-5080]^§^ | *0.680* | ***<0.001*** | ***0.003*** |
| Not aerated lung tissue (g)  PEEP 5 cmH_2_O  PEEP 45 cmH_2_O | 503 [451-625]  225[100-288]^§^ | 434 [359-624]  321 [255-488]^§°^ | *0.729* | ***<0.001*** | ***<0.001*** |
| Poorly aerated lung tissue (g)  PEEP 5 cmH_2_O  PEEP 45 cmH_2_O | 232 [215-308]  229 [192-256] | 304 [227-368]  229 [176-294]^§^ | *0.565* | ***<0.001*** | *0.279* |
| Well aerated lung tissue (g)  PEEP 5 cmH_2_O  PEEP 45 cmH_2_O | 368 [281-459]  664 [617-720]^§^ | 409 [293-492]  527 [437-641]^§°^ | *0.354* | ***<0.001*** | ***0.002*** |
| Over aerated lung tissue (g)  PEEP 5 cmH_2_O  PEEP 45 cmH_2_O | 1 [0-5]  77 [30-116]^§^ | 1 [0-8]  50 [18-90]^§^ | *0.945* | ***<0.001*** | *0.413* |
| Not aerated lung tissue (%)  PEEP 5 cmH_2_O  PEEP 45 cmH_2_O | 46 [39-51]  18 [10-23]^§^ | 40 [31-47]  29 [23-40]^§°^ | *0.417* | ***<0.001*** | ***<0.001*** |
| Poorly aerated lung tissue (%)  PEEP 5 cmH_2_O  PEEP 45 cmH_2_O | 20 [18-25]  19 [16-22] | 24 [21-28]  19 [16-21]^§^ | *0.561* | ***<0.001*** | *0.125* |
| Well aerated lung tissue (%)  PEEP 5 cmH_2_O  PEEP 45 cmH_2_O | 31 [31-34]  60 [46-62]^§^ | 34 [28-42]  46 [37-51]^§°^ | *0.238* | ***<0.001*** | ***<0.001*** |
| Over aerated lung tissue (%)  PEEP 5 cmH_2_O  PEEP 45 cmH_2_O | 0 [0-0]  7 [3-9]^§^ | 0 [0-1]  4 [1-7]^§^ | *0.691* | ***<0.001*** | *0.262* |

**Table 5S. Respiratory mechanics, gas exchange and quantitative radiological characteristics at two levels of airways pressure in FOCAL pattern group divided according to the potential recruitment. Two-way repeated measures ANalysis Of VAriance followed by all pairwise multiple comparison procedures (Holm-Šidák method) were used.**

**§: significant post-hoc comparison with PEEP 5 cmH_2_O;**

**°: significant post-hoc comparison with RECRUITERS group.**

| FOCAL PATTERN | Recruiters  (n=13) | Non Recruiters  (n=45) | *p* |
| --- | --- | --- | --- |
| Total lung gas (mL) | 1266 [927-1538] | 1192 [916 – 1994] | *0.970* |
| Total lung weight (g) | 1158 [1074 - 1237] | 1242 [1033 - 1398] | *0.685* |
| Total lung volume (mL) | 2355 [2227 - 2775] | 2574 [1997 - 3346] | *0.854* |
| Not aerated lung tissue (g) | 503 [451 - 625] | 434 [359 - 624] | *0.208* |
| Poorly aerated lung tissue (g) | 232 [215 - 308] | 304 [227 - 368] | *0.355* |
| Well aerated lung tissue (g) | 392 ± 111 | 419 ± 148 | *0.494* |
| Over aerated lung tissue (g) | 1 [0 - 5] | 1 [0 – 8] | *0.723* |
| Δ_45-5_ total lung gas (mL) | 2245 ± 521 | 1631 ± 568 | ***<0.001*** |
| Δ_45-5_ total lung volume (mL) | 2208 ± 567 | 1634 ± 588 | ***0.003*** |
| Δ_45-5_ not aerated lung tissue (g) | -317 [-432- - -226] | -100 [-131- -68] | ***<0.001*** |
| Δ_45-5_ poorly aerated lung tissue (g) | -28 [-65 - 14] | -67 [-128 - - 19] | 0.170 |
| Δ_45-5_ well aerated lung tissue (g) | 261 [121 – 392] | 128 [49-165] | *0.013* |
| Δ_45-5_ over aerated lung tissue (g) | 73 [30 – 106] | 45 [18 -80] | *0.236* |
| Lung recruitment (%) | 24 [19 – 30] | 9 [6 – 11] | **<0.001** |
| Overinflation (%) | 6.5 [2.6 – 9.6] | 3.8 [1.8 – 7.5] | 0.304 |

**Table 6S. Quantitative radiological characteristics at PEEP 5 cmH_2_O and between two levels of airways pressure in the focal group according to the potential recruitment. Continuous data are expressed as mean (SD) or median [interquartile range]. Student t test or Mann-Whitney rank-sum test, were used as appropriate.**

| DIFFUSE PATTERN | Recruiters  (n=71) | Non Recruiters  (n=39) | *p* |
| --- | --- | --- | --- |
| Age (years) | 58 ± 17 | 54 ± 16 | *0.227* |
| Male sex [% (number)] | 65 (46) | 79 (31) | *0.164* |
| Weight (kg) | 75 [60 – 85] | 75 [60 – 83] | *0.993* |
| BMI (kg/m^2^) | 25 [22 – 28] | 25 [22 – 30] | *0.502* |
| Origin of ARDS  Pulmonary [% (n)]  Extrapulmonary [% (n)] | 80 (57)  20 (14) | 56 (22)  44 (17) | ***0.015*** |
| ARDS severity  Mild [% (n)]  Moderate [% (n)]  Severe [% (n)] | 3 (2)  53 (38)  44 (31)* | 15 (6)  67 (26)  18 (7)* | ***0.004*** |
| SAPS II | 40 [34 – 51] | 35 [28 – 49] | *0.202* |
| Tidal volume (mL) | 484 ± 109 | 491 ± 86 | *0.732* |
| Tidal volume per ideal body weight (mL/kg) | 7.4 [6.7 – 8.3] | 7.3 [6.6 – 8.1] | *0.591* |
| Respiratory rate (breath for minute) | 16 [14 – 20] | 16 [15 – 19] | *0.713* |
| Minute ventilation (L/min) | 8.2 ± 2.6 | 9.0 ± 2.2 | *0.100* |
| Plateau pressure (cmH_2_O) | 19 ± 4 | 19 ± 4 | *0.409* |
| Driving pressure (cmH_2_O) | 14 ± 4 | 14 ± 4 | *0.659* |
| Respiratory system elastance (cmH_2_O/L) | 28 [24 – 36] | 27 [22 – 34] | *0.412* |
| Chest wall elastance (cmH_2_O/L) | 5 [3 – 8] | 6 [4 - 8] | *0.252* |
| Lung elastance (cmH_2_O/L) | 23 [18 – 28] | 20 [15 – 24] | *0.112* |
| PaCO_2_ (mmHg) | 49 [43 – 54] | 46 [42 – 52] | *0.328* |
| PaO_2_ (mmHg) | 64 [57 – 73] | 68 [63 – 81] | ***0.067*** |
| PaO_2_/FiO_2_ | 104 [75 – 139] | 131 [106 – 168] | ***0.001*** |
| Physiological dead space | 0.68 ± 0.13 | 0.64 ± 0.11 | *0.104* |
| ICU length of stay, days | 18 [11 – 25] | 25 [11 – 36] | *0.193* |
| ICU mortality  Dead  Alive | 46 (32)  54 (37) | 44 (17)  56 (22) | *0.938* |

**Table 7S. Diffuse population characteristics at PEEP 5 cmH_2_O according to the potential recruitment**. **Continuous data are expressed as mean (SD) or median [interquartile range], while categorical data are expressed as % (number). Student t test or Mann-Whitney rank-sum test and chi-square or Fisher exact test, were used as appropriate.**

| DIFFUSE PATTERN | Recruiters  (n=71) | Non Recruiters  (n=39) | *p* |
| --- | --- | --- | --- |
| Δ_15-5_ End-inspiratory airway pressure (cmH_2_O) | 8 [7 – 11] | 9 [8 – 11] | *0.152* |
| Δ_15-5_ Driving Pressure (cmH_2_O) | -1 [-2 – 1] | 0 [-2 – 1] | *0.500* |
| Δ_15-5_ Respiratory system elastance (cmH_2_O/L) | -2 [-5 – 2] | 0 [-3 – 1] | *0.622* |
| Δ_15-5_ Lung elastance (cmH_2_O/L) | -1 [-5 – 3] | 0 [-5 - 2] | *0.857* |
| Δ_15-5_ Chest wall elastance (cmH_2_O/L) | 1 ± 3 | 0 ± 3 | *0.222* |
| Δ_15-5_ PaCO_2_ (mmHg) | 0 [-2 – 2] | 0 [-2 – 2] | *0.884* |
| Δ_15-5_ PaO_2_/FiO_2_ | 58 [32 – 108] | 62 [30 – 95] | *0.672* |
| Δ_15-5_ Physiological dead space | 0.00 ± 0.04 | 0.01 ± 0.03 | *0.576* |

**Table 8S. Changes in respiratory mechanics and gas exchange at 2 levels of PEEP in diffuse group according to the potential recruitment.**

**Quantitative data are expressed as mean (SD) or median [interquartile range; Student t test or Mann-Whitney rank-sum test, as appropriate, have been used for continuous variable analysis.**

| DIFFUSE PATTERN | Recruiters  (n=71) | Non Recruiters  (n=39) | *p_GROUP_* | *p_PEEP_* | *p_GROUP X PEEP_* |
| --- | --- | --- | --- | --- | --- |
| End-inspiratory airway pressure (cmH_2_O)  PEEP 5 cmH_2_O  PEEP 15 cmH_2_O | 19 [17 – 21]  28 [25 – 30]^§^ | 18 [16 – 20]  28 [24 – 30]^§^ | *0.662* | ***<0.001*** | *0.291* |
| Driving Pressure (cmH_2_O)  PEEP 5 cmH_2_O  PEEP 15 cmH_2_O | 14 [11-16]  13 [11-16] | 13 [11-16]  14 [11-16] | *0.825* | *0.184* | *0.562* |
| Respiratory system elastance (cmH_2_O/L)  PEEP 5 cmH_2_O  PEEP 15 cmH_2_O | 28 [24-36]  27 [31-33] | 27 [22-34]  29 [21-32] | *0.501* | *0.245* | *0.804* |
| Lung elastance (cmH_2_O/L)  PEEP 5 cmH_2_O  PEEP 15 cmH_2_O | 23 [19-28]  21 [16-26] | 21 [17-27]  20 [17-25] | *0.316* | *0.291* | *0.840* |
| Chest wall elastance (cmH_2_O/L)  PEEP 5 cmH_2_O  PEEP 15 cmH_2_O | 6 [3-8]  6 [4-8] | 6 [5-8]  7 [5-8] | *0.827* | *0.868* | *0.169* |
| PaCO_2_ (mmHg)  PEEP 5 cmH_2_O  PEEP 15 cmH_2_O | 49 [43-54]  49 [42-55] | 46 [42-52]  45 [42-52] | *0.739* | *0.683* | *0.828* |
| PaO_2_/FiO_2_ (mmHg)  PEEP 5 cmH_2_O  PEEP 15 cmH_2_O | 104 [75-139]  176 [137-231]^§^ | 131 [106-169]°  204 [153-256]^§^ | ***0.015*** | ***<0.001*** | *0.349* |
| Physiological dead space  PEEP 5 cmH_2_O  PEEP 15 cmH_2_O | 0.68 [0.62-0.74]  0.65 [0.61-0.70] | 0.64 [0.58-0.71]  0.62 [0.57-0.72] | *0.127* | *0.589* | *0.588* |
| Total lung gas (mL)  PEEP 5 cmH_2_O  PEEP 45 cmH_2_O | 657 [480-852]  1962 [1467-2609]^§^ | 924 [674-1529]°  2269 [1755-2815]^§^° | ***0.002*** | ***<0.001*** | ***0.444*** |
| Total lung weight (g)  PEEP 5 cmH_2_O  PEEP 45 cmH_2_O | 1660 [1381-2092]  1667 [1438-2038] | 1541 [1390-1948]  1594 [1378-1963] | *0.346* | *0.105* | *0.567* |
| Total lung volume (mL)  PEEP 5 cmH_2_O  PEEP 45 cmH_2_O | 2377 [2088-2694]  3751 [3103-4584]^§^ | 2768 [2240-3274]  4074 [3566-4666]^§^ | *0.124* | ***<0.001*** | *0.531* |
| Not aerated lung tissue (g)  PEEP 5 cmH_2_O  PEEP 45 cmH_2_O | 977 [652-1287]  477 [229-695]^§^ | 614 [437-967]°  461 [331-779]^§^ | *0.211* | ***<0.001*** | ***<0.001*** |
| Poorly aerated lung tissue (g)  PEEP 5 cmH_2_O  PEEP 45 cmH_2_O | 508 [365 -692]  624 [398-867]^§^ | 496 [415-678]  437 [277-599]^§^° | ***0.039*** | *0.412* | ***<0.001*** |
| Well aerated lung tissue (g)  PEEP 5 cmH_2_O  PEEP 45 cmH_2_O | 205 [151-271]  578 [453- 729]^§^ | 334[224-528]°  626 [553-745]^§^ | ***0.001*** | ***<0.001*** | ***0.001*** |
| Over aerated lung tissue (g)  PEEP 5 cmH_2_O  PEEP 45 cmH_2_O | 0 [0-0]  5 [1-13]^§^ | 0 [0-2]  10 [1-18]^§^° | *0.107* | ***<0.001*** | *0.216* |
| Not aerated lung tissue (%)  PEEP 5 cmH_2_O  PEEP 45 cmH_2_O | 58 [47-67]  27 [16 -36]^§^ | 42 [27-53]°  29 [19-41]^§^ | *0.109* | ***<0.001*** | ***<0.001*** |
| Poorly aerated lung tissue (%)  PEEP 5 cmH_2_O  PEEP 45 cmH_2_O | 31 [21-39]  35 [28-46]^§^ | 32 [27 -41]  25 [20-36]^§^° | *0.074* | *0.554* | ***<0.001*** |
| Well aerated lung tissue (%)  PEEP 5 cmH_2_O  PEEP 45 cmH_2_O | 12 [8-15]  33 [25 -43]^§^ | 19 [15-29]°  42 [32-46]^§^ | ***<0.001*** | ***<0.001*** | ***0.004*** |
| Over aerated lung tissue (%)  PEEP 5 cmH_2_O  PEEP 45 cmH_2_O | 0 [0-0]  0 [0-1]^§^ | 0 [0-0]  1 [0-1]^§^ | *0.153* | ***<0.001*** | ***0.339*** |

**Table 9S. Respiratory mechanics, gas exchange and quantitative radiological characteristics at two levels of airways pressure in DIFFUSE pattern group divided according to the potential recruitment. Two-way repeated measures ANalysis Of VAriance followed by all pairwise multiple comparison procedures (Holm-Šidák method) have been used for analysis.**

**§: significant post-hoc comparison with PEEP 5 cmH_2_O;**

**°: significant post-hoc comparison with RECRUITERS group.**

| DIFFUSE PATTERN | Recruiters  (n=71) | Non Recruiters  (n=39) | *p* |
| --- | --- | --- | --- |
| Total lung gas (mL) | 657 [480 – 852] | 924 [674 – 1529] | ***<0.001*** |
| Total lung weight (g) | 1660 [1381 - 2092] | 1541 [1390 - 1948] | *0.320* |
| Total lung volume (mL) | 2377 [2088 – 2694] | 2768 [2240 - 3274] | ***0.021*** |
| Not aerated lung tissue (g) | 977 [652 – 1287] | 614 [437 – 967] | ***<0.001*** |
| Poorly aerated lung tissue (g) | 508 [365 – 692] | 496 [415 – 678] | *0.764* |
| Well aerated lung tissue (g) | 213 ± 104 | 373 ± 189 | ***<0.001*** |
| Over aerated lung tissue (g) | 0 [0 – 0] | 0 [0 – 2] | *0.374* |
| Δ_45-5_ total lung gas (mL) | 1369 ± 561 | 1283 ± 562 | *0.445* |
| Δ_45-5_ total lung volume (mL) | 1381 ± 598 | 1307 ± 571 | *0.526* |
| Δ_45-5_ not aerated lung tissue (g) | -487 [-618 - -357] | -171 [-211 - -101] | ***<0.001*** |
| Δ_45-5_ poorly aerated lung tissue (g) | 127 [-35 – 288] | -97 [-188 - -31] | ***<0.001*** |
| Δ_45-5_ well aerated lung tissue (g) | 386 ± 167 | 281 ± 137 | ***<0.001*** |
| Δ_45-5_ over aerated lung tissue (g) | 5 [1 – 12] | 8 [1 – 17] | *0.274* |
| Lung recruitment (%) | 27 [22 – 34] | 10 [8 – 14] | **<0.001** |
| Overinflation (%) | 0.3 [0.0 – 0.8] | 0.5 [0.1 – 1.2] | 0.223 |

**Table 10S. Quantitative radiological characteristics at PEEP 5 cmH_2_O and between two levels of airways pressure in the diffuse group according to the potential recruitment. Continuous data are expressed as mean (SD) or median [interquartile range], while categorical data are expressed as % (number). Student t test or Mann-Whitney rank-sum test and chi-square or Fisher exact test, were used as appropriate.**
